# Investigation on Microstructure and Properties of Duplex Stainless Steel Welds by Underwater Laser Welding with Different Shielding Gas

**DOI:** 10.3390/ma14174774

**Published:** 2021-08-24

**Authors:** Kai Wang, Changlei Shao, Xiangdong Jiao, Jialei Zhu, Zhihai Cai, Congwei Li

**Affiliations:** 1 School of Mechanical Engineering, Beijing University of Chemical Technology, Beijing 100029, China; 18810660456@163.com (K.W.); jiaoxiangdong@bipt.edu.cn (X.J.); 2 Shanghai Nuclear Engineering Research & Design Institute, Shanghai 200233, China; shaocl@snerdi.com.cn; 3 Beijing Institute of Petrochemical Technology, Beijing 102617, China; m17854117942@163.com; 4 Army Academy of Armored Forces, Beijing 100072, China; caizhihai2052@163.com

**Keywords:** duplex stainless steel, underwater laser wire filling welding, hyperbaric chamber, intermetallic inclusions, pitting corrosion resistance

## Abstract

Taking S32101 duplex stainless steel as the research object, underwater laser wire filling welding technology was used for U-groove filling welding. The influence of different shielding gas compositions on the ferrite content, microstructure, mechanical properties and pitting corrosion resistance was studied by simulating a water depth of 15 m in the hyperbaric chamber. The results show that, under the same process parameters, the size and proportion of austenite in the weld when using pure nitrogen as the shielding gas are larger than those protected by other shielding gases. In a mixed shielding gas, the increase in nitrogen content has little effect on the strength and toughness of the weld. Regardless of the shielding gas used, the base metal was the weakest part of the weld. At the same time, intermetallic inclusions have an adverse effect on the impact toughness of the weld. The pitting corrosion resistance of the welds depends on the Cr_2_N content in the heat-affected zone. The precipitation and enrichment of Cr_2_N causes local chromium deficiency, which is the main factor for the weak pitting corrosion ability of the heat-affected zone. Pure nitrogen protection has a better corrosion resistance than other gas protection.

## 1. Introduction

Duplex stainless steel (DSS) has been widely developed in recent years because of its good corrosion resistance and economic benefits. Due to both its dual-phase equilibrium microstructure of ferrite and austenite and high content of alloy elements, DSS has a high strength and toughness and excellent corrosion resistance [1,2]. Therefore, DSS is increasingly used as structural materials in nuclear power plants, offshore engineering construction and the petrochemical industry [3]. In order to reduce the cost of operation and maintenance, underwater welding is usually used to repair cracks and surface corrosion defects in nuclear power plants. Underwater welding is mainly divided into three methods: underwater dry welding, underwater wet welding and underwater local dry welding [4,5,6,7] Underwater dry welding has good welding quality, but the welding process is complex, resulting in a significant increase in cost; the underwater wet welding device is simple, but it cannot guarantee the welding quality; local dry welding combines the advantages of the first two methods, which not only ensures the welding quality, but also reduces the equipment cost. It is very suitable for the construction [8] and maintenance of underwater nuclear power plants [4].

As a new welding technology, underwater laser welding (ULW) has gradually become the most promising maintenance technology for nuclear power plant underwater welding. Feng et al. studied the effects of protective materials and water on the repair quality of Ni-Al bronze by underwater laser welding [9]. Ning G. et al. proved the effect of water depth on the weld quality and welding process in underwater laser welding [10]. At the same time, Ning G. carried out welding experiments on a Ti-6Al-4V alloy with filler wire underwater laser beam welding (ULBW) technology, and obtained joints close to the welding quality in air [11]. Tomków et al. completed the butt welding experiment of high-strength low-alloy s460n steel by using a local cavity method, and tested the mechanical properties of the welded joints in order to obtain a high quality [12]. Based on the existing research results, the microstructure and properties of underwater laser welding of duplex stainless steel were studied using the local cavity method.

In DSS laser welding, the proportion of the equilibrium phase is very important; the equal fraction of ferrite and austenite is the best combination of mechanical properties and corrosion resistance [13]. ULW technology has a high applicability in the field of nuclear power plant repair. However, some welding metallurgical challenges limit its applications in underwater maintenance. The metallurgical solidification of DSS is fully ferritic, followed by the transformation from solid-state ferrite to austenite controlled by thermal cycle diffusion. When laser welding is carried out in an underwater environment, the rapid cooling rate largely suppresses the formation of austenite and restricts the optimum phase equilibrium in DSS.

Nitrogen is a strong austenite-forming element because of its high diffusion rate [14,15]. When using ULW, the rapid heating and cooling cycle restricts the austenite formation and disturbs the optimum phase equilibrium in DSS, resulting in nitrogen atom loss and a high ferrite content [16], which is not conducive to the pitting corrosion resistance of welded joints. The decrease in nitrogen content is not limited to weld metal (WM): the heat-affected zone (HAZ) is also affected by the nitrogen diffusion from HAZ to WM [17,18,19].

According to Sakai et al., the austenite content of DSS should be higher than 50% in order to obtain high impact toughness [20]. Miura and Ogawa also pointed out that the lowest pitting corrosion rate is also associated with an austenite content of 50% or more [21].

In this paper, the effects of pure argon, pure nitrogen and different proportions of the nitrogen–argon mixture as a shielding gas on the mechanical properties and pitting corrosion resistance of DSS welded joints were studied, and the influence mechanism was analyzed.

## 2. Experimental Procedure

### 2.1. Equipment and Materials

**Figure 1:**
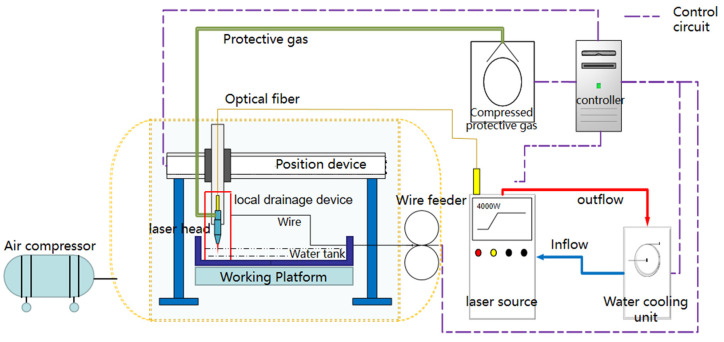
Schematic diagram of underwater laser welding test system in hyperbaric environment.

**Figure 2:**
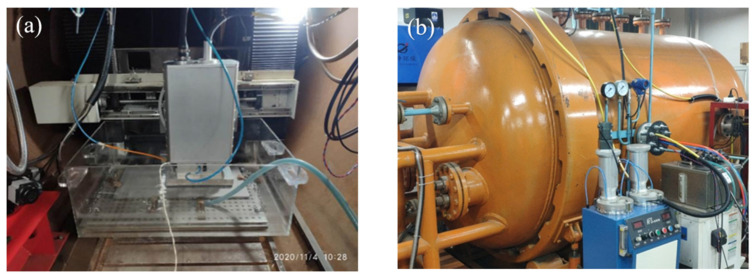
Hyperbaric underwater laser welding test system: (**a**) underwater laser welding test platform; (**b**) hyperbaric test chamber.

The ULW was performed using RCL-C6000 fiber laser of Ruike (specifications given in Table 1), with a maximum output power of 6 kW and wavelength of 1070 nm. As shown in Figure 1, the experimental system is mainly composed of two parts: the hyperbaric experimental chamber and the ULW experimental platform, which can meet the requirements of rapid and accurate positioning of underwater welding and simulation of pressure environment. During the welding process, the pressure of the cabin was kept at 0.15 MPa by filling compressed gas to simulate the water depth of 15 m. The wet underwater environment was simulated by injecting water into the experimental tank, and the water depth in the tank was 30 mm. A sealed underwater laser head and local drainage device were developed. The laser beam was transmitted to the local dry space inside the local drainage device through optical fiber, and the ULW repair was realized by laser wire filling technology. Figure 2a,b was underwater laser welding test platform and hyperbaric test chamber respectively.

**Figure 3:**
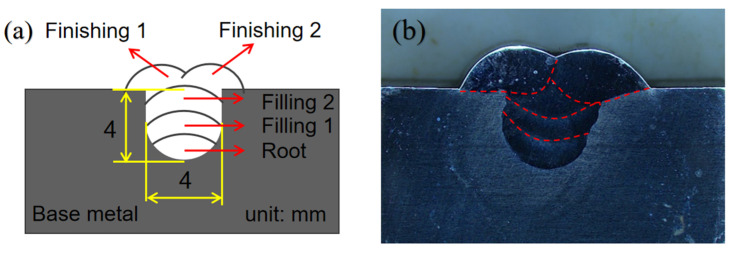
U-groove design and cross-sectional appearance of (**a**) the welded joint and (**b**) the schematic diagram.

S32101 DSS was used as the base metal, and the dimensions of test plates were prepared with 600 mm × 300 mm × 25.8 mm. The design of U-groove filling joint is shown in Figure 3a,b, as part of the weld section. The chemical compositions of the BM and filler metal are summarized in Table 2. The mechanical properties of the base metal are shown in Table 3. The chemical composition and mechanical properties are provided by Anshan Iron and Steel Group Co., Ltd., located in Liaoning, China.

### 2.2. Welding Process

In 0.15 MPa pressure, the welding process of U-type groove with laser wire filling was carried out. The microstructure and mechanical properties of underwater welds with different shielding gas were studied. The process parameters are shown in Table 4.

The microstructure of weld specimens was characterized by optical microscopy (OM) and scanning electron microscopy (SEM) combined with energy dispersive spectroscopy (EDS). MTS E45 testing machine was used for tensile test, and the dimensions of the tensile specimens were prepared in accordance with ASTM E-8 standard. Charpy V-notch impact tests of the WM and HAZ were performed at −40 °C, and the impact specimens were prepared in accordance with ASTM A370 (2017). The microhardness was measured from HAZ to WM on a Vickers hardness tester. The test load was 500 g and the dwell time was 10 s. The percentage elongation, tensile and hardness were determined at ambient temperature.

### 2.3. Electrochemical Corrosion Tests

The evaluation of weld pitting sensitivity by potentiodynamic tests has been confirmed as a time-saving and non-destructive method [22]. A potentiostat with a three-electrode cell was used, with saturated calomel electrode (SCE) and platinum electrode as reference electrode and counter electrode, respectively, and the test piece as working electrode. The potentiodynamic test was carried out in 1 m NaCl solution at 60 °C. The N_2_ purging was carried out for 30 min for deoxidization before each test.

The specimens were composed of WM and HAZ with an exposed area of 25 mm^2^. In order to reduce the effect of the working electrode surface condition on the test result, the specimens were grounded to 2000 grit and polished with diamond paste.

In order to improve the accuracy of the experiment, the working electrode was cathodically polarized at room temperature for 10 min. When the steady-state open circuit potential (E_ocp_) was reached (approximately 10 min), the experiment was started and the specimen was anodically polarized up to the potential of +300 mV_SCE_.

## 3. Results and Discussion

### 3.1. Microstructural Characterization

**Figure 4:**
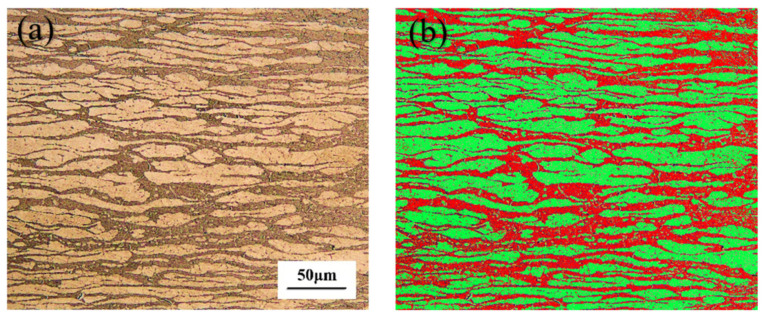
(**a**) Microstructure of the BM; (**b**) phases volume fraction of the BM.

The microstructure of BM is presented in Figure 4a. Figure 4b displays the two phases volume fractions, confirmed as almost equal (46 ± 1 vol.% Ferrite), in the microstructure measured by the software Axio vert.a1 configured by an optical microscope (OM, Zeiss). The austenite (light phase) is distributed as laths in the ferrite matrix. However, in the laser welding process, the nitrogen in the molten pool can be lost and the austenite content of the weld is reduced [23].

On the basis of the DeLong diagram, the American Welding Society (AWS) recommends the WRC~92 structure diagram, where nitrogen is a strong austenite-forming element [24]. It can not only improve the strength of DSS, but also increase the toughness, reduce the formation of the harmful intermetallic phase and reduce the tendency of the precipitated phase, due to a high chromium and molybdenum content.

Therefore, this paper investigates the effect of nitrogen on the microstructure and mechanical properties of DSS-welded joints by underwater laser wire filling welding.

**Figure 5:**
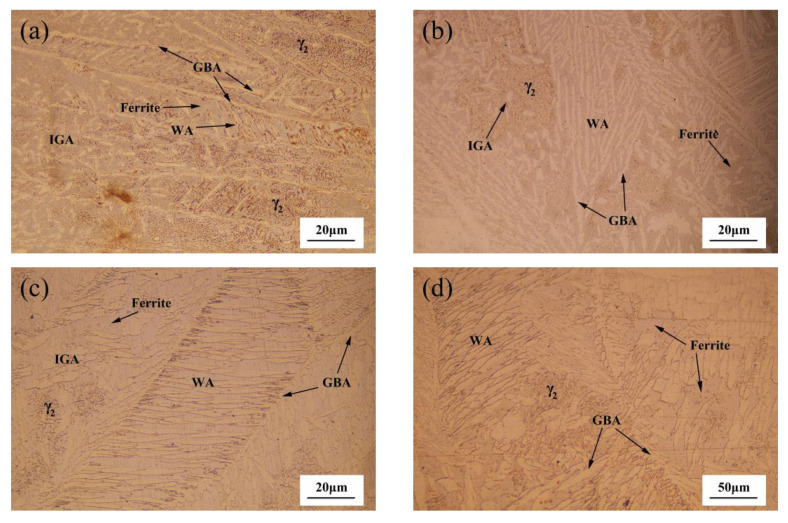
Weld microstructure: (**a**) sample A in pure Ar shielding gas; (**b**) sample B in 90% Ar + 10% N_2_ hybrid shielding gas; (**c**) sample C in 50% Ar + 50% N_2_ hybrid shielding gas; (**d**) sample D in pure N_2_ shielding gas.

**Figure 6:**
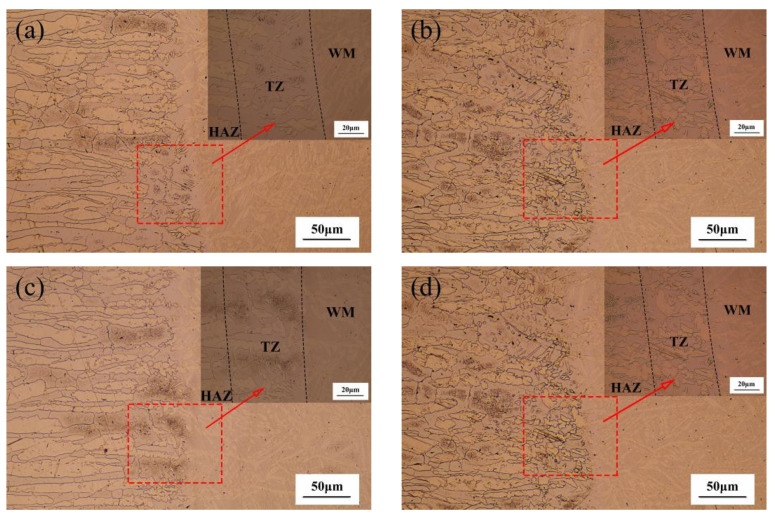
HAZ microstructure: (**a**) sample A in pure Ar shielding gas; (**b**) sample B in 90% Ar + 10% N_2_ hybrid gas; (**c**) sample C in 50% Ar + 50% N_2_ hybrid gas; (**d**) sample D in pure N_2_ shielding gas.

Rapid cooling will restrict the formation of austenite and disturb the optimal phase balance in DSS. In Amir Baghdadhi’s experiments, laser reheating is used to avoid low austenite fraction and nitride formation [25]. Compared with air, the cooling rate of the molten pool of underwater laser welding is faster and the temperature gradient is larger. Therefore, in the experiment, the method of reducing the welding speed was used to improve the welding heat input, and a microstructure similar to that in air was obtained. Figure 5 presents the microstructure of S32101 stainless steel welds under different shielding gas compositions. Figure 6a–d are the weld microstructure of samples A, B, C and D, respectively. It is observed that the microstructure is mainly composed of two phases: the light phase is austenite and the dark phase is ferrite. However, compared with the base metal, there are significant differences in the morphology of the γ-phases formed in the grain boundaries of the α-phase in the weld zone. Moreover, the recrystallized size of the γ-phase in the A and B specimens is coarser than that in the C and D specimens. This is due to the austenite transformation of weld metal under the action of a welding thermal cycle during underwater welding. The previous-transformation austenite (PTA) and grain boundary austenite (GBA) began precipitating along the ferrite grain boundaries. A large amount of austenite precipitated as Widmanstätten austenite (WA) and grew into austenite grains in the ferrite. At the same time, a large number of intragranular austenite (IGA) particles with an acicular morphology were observed within the ferrite grains. Compared with the welds prepared with argon as a shielding gas, the volume of WA, IGA and GBA of those prepared with the nitrogen mixture gas or pure nitrogen were increased, and the three types of austenite were coarsened (Figure 5d). However, the type of austenite in the weld did not change obviously under the different shielding gas conditions. Therefore, increasing the proportion of nitrogen in the shielding gas can significantly increase the austenite content, but the transformation type of the austenite was not affected.

Figure 6 shows the effects of different shielding gas compositions on the microstructure in the HAZs. The microstructure near the fusion line was composed of WM, a transition zone (TZ) and HAZ. Due to fact that the temperature of HAZ near the fusion line can increase to approximately 1000 °C, all of the γ-phases of the original duplex microstructures will further evolve. In the subsequent cooling process, some ferrite transforms into austenite, but the growth of the austenite grains was inhibited due to the decrease in the high temperature residence time in the underwater environment. Therefore, the HAZ consists of a large number of coarse polygonal ferrite and irregular austenite. It is also observed that the width of the polygonal ferrite band was approximately 50 μm. However, the microstructure of TZ was quite different under different shielding gas conditions. It can be seen that the more nitrogen content in the shielding gas, the more austenite content and the more uniform the distribution. In addition, due to the composition gradient and diffusion effect of the weld and HAZ, the concentrations of Cr and Ni in HAZ near the fusion line were decreased, thus the γ-phase began to nucleate in the ferrite or dislocation line. However, due to the increase in undercooling, the γ-phase formed is very small, which is different from the weld γ-phase, and has little difference with ferrite in composition, and so belongs to non-diffusion transformation.

**Figure 7:**
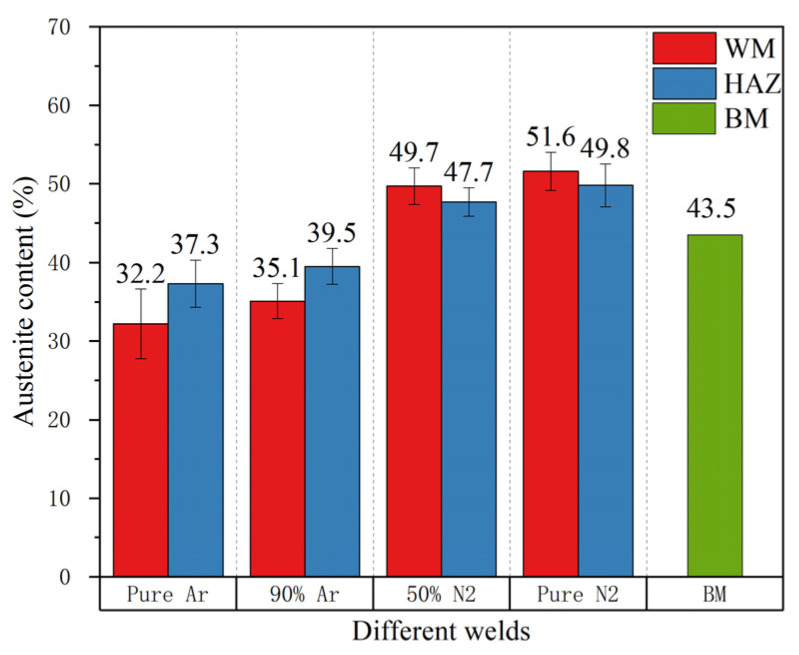
The effect of the shielding gas composition on the austenite fractions of the DSS welds.

Figure 7 shows the austenite content of four kinds of welds in different shielding gas compositions and BM. As can be seen from Figure 7, with the increase in the nitrogen content in the shielding gas, the content of austenite in WM and HAZ gradually increases. In addition, when the nitrogen content in the shielding gas increases to 50%, the austenite content increases greatly, and when the shielding gas is pure nitrogen, the increase range of the austenite content decreases. The reason for this phenomenon may be because the nitrogen element in the WM reached the saturation state at the austenite transformation temperature, and then reached the maximum value of the α/γ transformation rate. However, there was an obvious temperature gradient in HAZ near the fusion line, and the solubility of nitrogen in ferrite decreases rapidly with the decrease in temperature, so limited austenization occurred in the HAZs.

### 3.2. Mechanical Performance Testing

**Figure 8:**
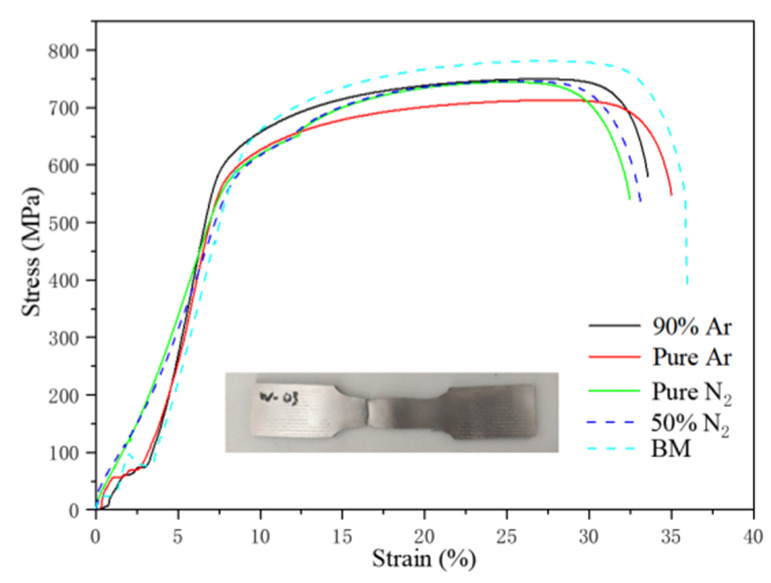
Stress–strain curve.

The stress–strain curves and tensile test results of the BM and welded joints are presented in Figure 8 and Table 5. Compared to the BM, the joints had a higher tensile strength and yield strength but their elongation was slightly lower than that of BM. The experimental results also present that the increase in nitrogen content in the shielding gas did not affect the strength and toughness of the joint. The four kinds of welded joints were all strengthened, and the BM was the weakest part of the joint.

There are three main reasons, which are as follows:

- The ferrite and austenite in the BM are banded along the rolling direction, whereas the ferrite and austenite in the WM and HAZ are interlaced with different directions, and the grain boundaries are increased, which can lock the dislocation and strengthen the joint;
- The atoms of Cr, Mo and Ni in the weld metal can be remelted at a high temperature, which can replace Fe atoms in the lattice and disturb the original lattice arrangement, and can also make dislocation movement difficult and strengthen the joint. As in HSLA steel, the addition of Mn and other alloying elements, such as copper (Cu), titanium (TI) and vanadium (V), both provide strengthening and an obtain ideal microstructure [26,27];
- The nitrogen atoms in the shielding gas are intercalated into the lattice in the form of an interstitial solid solution. The strengthening effect of the interstitial solid solution is more obvious than that of the replacement solid solution. The nitrogen atoms are mainly concentrated in the austenite phase and directly strengthen the austenite. Therefore, the strength performance of WM is better than that of BM.

### 3.3. Charpy V-Notch Impact Tests

**Figure 9:**
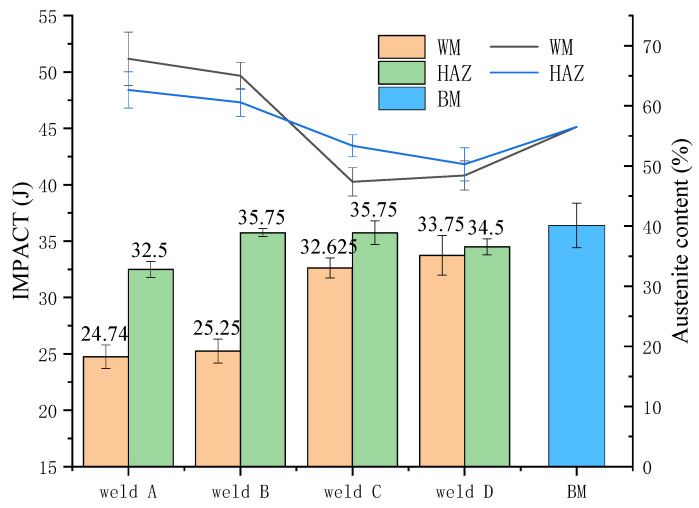
Austenite fractions and absorbing energy of the WMs in different shielding gas.

Charpy V-notch impact tests were performed on the weld zone and HAZ at −40 °C and the results are shown in Figure 9. In order to explain the relationship between impact energy and ferrite content, the ferrite content in different areas of weld is expressed by a polyline diagram. In the figure, the average absorbing energy of the WM of the A and B samples at −40 °C were 24.74 J and 25.25 J, respectively, which is merely 62.5% of the BM, with an average value of 40 J. The average absorbed energy of the WM of the C and D samples increased to 32.62 J and 33.75 J, which is approximately 82.5% of the BM. Compared with the BM, the average absorbing energy of the underwater laser welds decreased, but the A and B samples exhibited a more distinct fall than that of C and D. The main reason is due to the change in austenite content in the weld. According to Karlsson et al., the preferential growth orientation [100] of ferrite is in conjunction with the easiest cleavage planes, which would seriously damage the resulting toughness below the ductile-to-brittle transition temperature [28]. The change in HAZ impact energy under different shielding gas conditions is similar to that of the weld zone, which is also the result of the influence of the austenite content.

**Figure 10:**
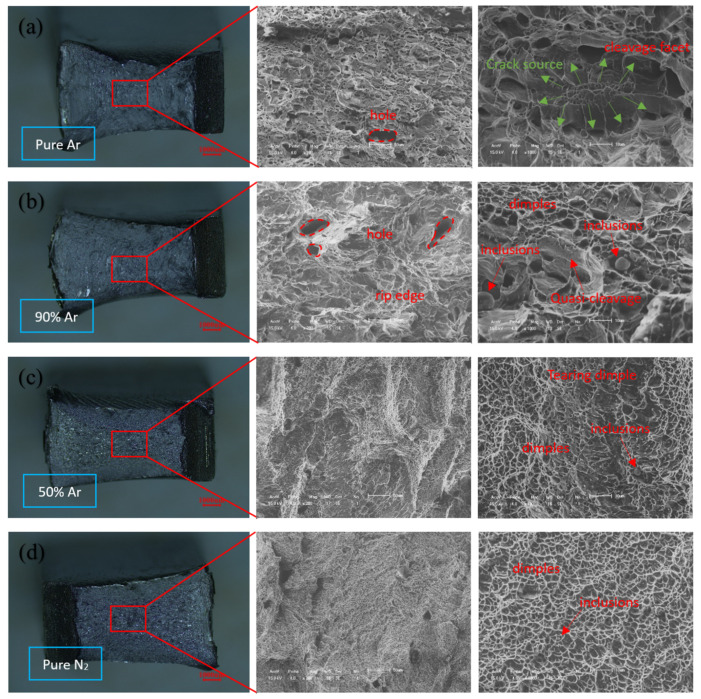
SEM micrographs showing the fracture surface morphology after Charpy impact testing at −40 °C: (**a**) the fractured surface of weld A; (**b**) the fractured surface of weld B; (**c**) the fractured surface of weld C; (**d**) the fractured surface of weld D.

Figure 10 shows the fractured surface morphologies after the impact tests at −40 °C. It can be seen that the fractured surface of welds A and B was relatively flat compared to that of welds C and D. The fractured surface is characterized by a quasi-cleavage plane, tearing ridge and fluvial pattern, which is consistent with the occurrence of a quasi-cleavage fracture. Some larger holes were also revealed in welds A and B, which may have resulted from the peeling of inclusions in the impact testing. At a higher magnification (1000×), a large number of high-density, short and curved tearing ridges were observed in weld A. The crack source radiated a fluvial pattern from the middle to the surrounding, and there were many micro-dimples around the crack source. Therefore, the quasi-cleavage fracture dominated the weld A fracture mode. The size of the deformation and the amount of dimples in weld B were larger than those in weld A, and some micro-holes caused by plastic deformation were also revealed around the quasi-cleavage small section, which indicates that the impact toughness of weld B is improved. Figure 10c depicts obvious tear dimples on the fracture surface of weld C, which indicates that the impact fracture at the weld joint appears as dimple ductile fracture characteristics. At the same time, there are obvious dimples and a fibrous network on the impact fracture surface of weld D, which is characterized by a ductile fracture.

Therefore, it can be concluded that the addition of nitrogen in the shielding gas can reduce the degree of the quasi-cleavage fracture, and that the size and quantity of nitride in the impact fracture were reduced. When the shielding gas changes from pure argon to pure nitrogen, the quasi-cleavage fracture transformed into ductile fracture.

**Figure 11:**
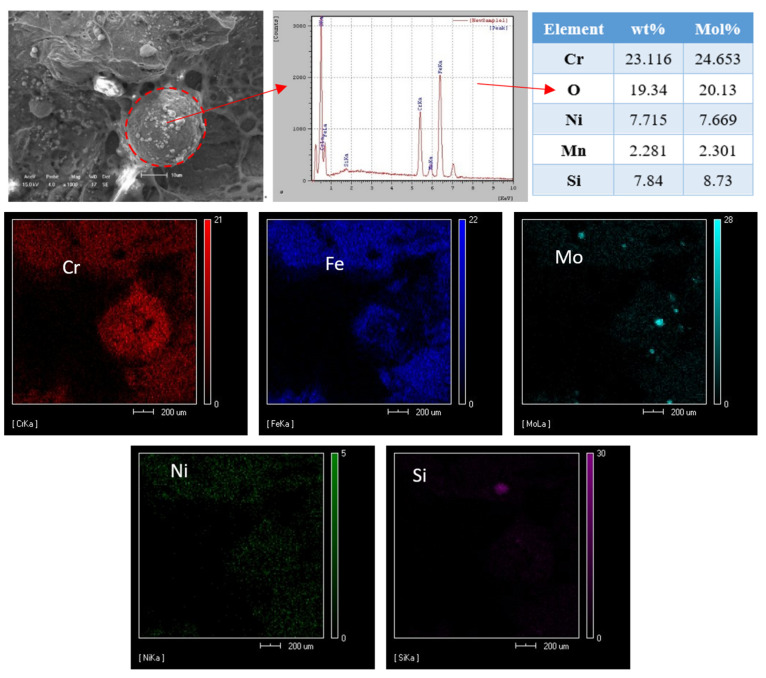
The results of EDS analysis of the inclusions in impact fracture surface.

The second-phase particles in the quasi-cleavage section were observed by high-magnification SEM, and the chemical composition and location of inclusions were measured by EDS (Figure 11). The results show that the inclusion was a Fe-Cr intermetallic compound. When the chromium content was high, the brittle and hard σ-phase begins to precipitate from δferrite at 820 °C. The σ-phase is a Fe-Cr intermetallic compound of WCR 45%, which will make the metal more brittle. On the other hand, the formation of inclusions will reduce the stress area, block the propagation path of plastic deformation and also reduce the impact toughness. Due to the σ-phase precipitating at the grain boundary, a large amount of chromium will be consumed, reducing the corrosion resistance of the material.

### 3.4. Hardness Tests

**Figure 12:**
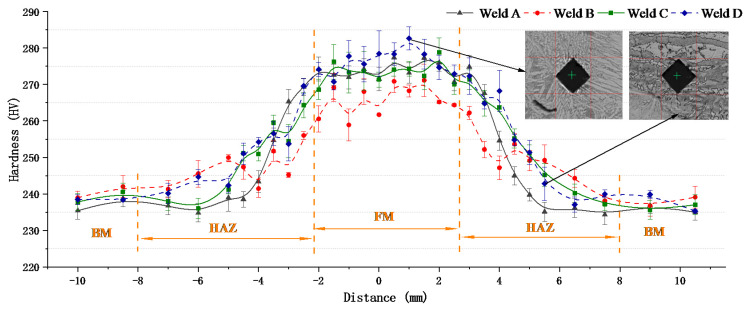
Micro-hardness measurements on the welded joints.

Figure 12 shows the change in microhardness in different weld cross sections. It can be seen that the average hardness of WM (average: ~280 HV) is higher than that of HAZ and BM (average micro-hardness: ~240 HV). The reason is that the higher Ni and Cr atoms in the weld can produce an obvious solid solution strengthening effect by remelting and replacing the iron atoms in the lattice. In addition, the ferrite and austenite in the weld metal interlace with each other, the number of grain boundaries increases and the direction is different, which leads to the increase in hardness. Weld B and weld C obtained similar micro-hardness values (275 HV), which were slightly lower than weld D (280 HV) and higher than weld A (270 HV). This may be due to the higher content of austenite in weld D, which leads to the increase in dislocation density and the increase in micro-hardness in the weld. However, the content of austenite in the argon-gas-protected weld is low, which leads to the decrease in the Cr element and the effect of solid solution strengthening, which, in turn, results in a decrease in hardness.

In addition, the dilution ratio of alloy elements also has an important influence on the microstructure and hardness.

**Figure 13:**
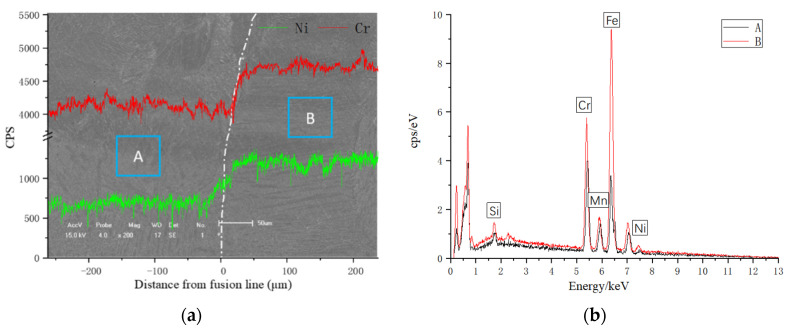
EDS result of line scanning for (**a**), and points scanning for (**b**); A, B points are HAZ and WM scanning zone by EDS.

In order to further determine the change in Ni and Cr elements from WM to HAZ near the fusion line, spot and line scanning by EDS were conducted from the HAZ to the side of the weld in Figure 13. The line scanning result is shown in Figure 13a, and the element compositions of points A and B are listed in Figure 13b. As can be seen in Figure 13a, the microhardness of the transition zone is significantly different from that of the weld zone and substrate. Table 6 shows the experimental results of EDS point scanning for four welds under different shielding gas conditions. Due to the fact that the change trend is similar, the EDS line scanning test is not repeated for the other three samples.

Due to the local composition gradient and the diffusion effect of alloy elements, there was an obvious concentration gradient between WM and HAZ. Since the transition zone had a considerable amount of Cr with a high affinity for carbon, the interstitial atoms of carbon migrated from the partially melted zone in HAZ during remelting. At this time, a carbon-poor ferritic band with a polygonal morphology was formed adjacent to the fusion boundary in the HAZ. Polygonal ferrite and irregular austenite were interlaced in order to increase dislocation energy, which is considered to be the major factor for the increase in hardness. The existence of the ferrite-banded region has been verified in many literatures. Some scholars call it the ferrite-stabilized region [29], carbon-poor region [30,31] and decarburized region [32]. It is reported [30] that this carbon-poor zone has a jagged morphology and its width depends upon both the temperature gradient and the carbon content of the substrate.

### 3.5. Potentiodynamic Polarization

**Figure 14:**
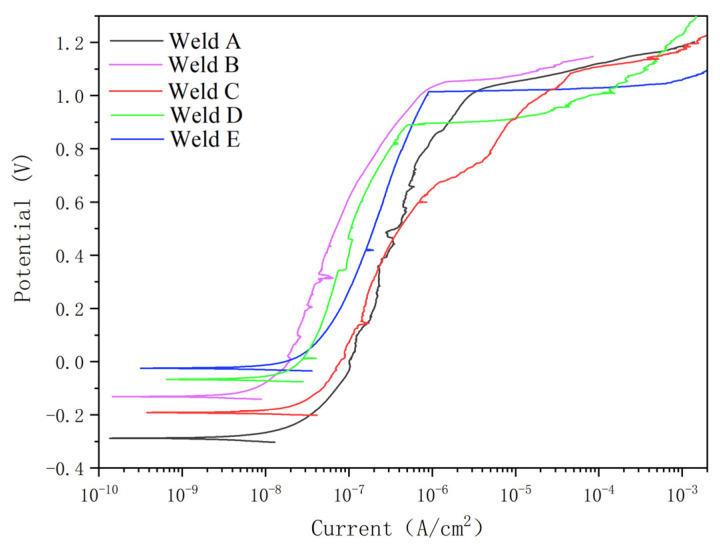
Anodic polarization curves of the DSS joints welded in different shielding gases.

In order to study the effect of N_2_ content in the protective gas on the pitting behavior of S32101 welds by ULW, the polarization test was conducted at 60 °C at the scanning rate of 1.67 mV/s in 1 m NaCl solution. Figure 14 shows the effects of different shielding gases on the anodic polarization responses of the DSS WM and BM. From the slope of the passivation current densities, it can be seen that, with the austenite phase increased, the specimen with pure N_2_ protection exhibited a lower corrosion current density range compared with that of the pure Ar protection. The difference in the responses to the localized corrosion was the result of the change in nitrogen content, because nitrogen promotes the formation of the austenite stability phase.

Table 7 shows the electrochemical parameter values in Figure 14. The transpassive potential (*E*_t_) determines the pitting corrosion susceptibility, and the passive current density (*i*_p_) determines the corrosion rates of the welded zone in the passivation range.

As shown in Table 7, the value of Et was increased significantly with the addition of nitrogen gas, and the value of ip was decreased. This means that the pitting corrosion resistance of the passive film is superior to that of pure Ar as a shielding gas. The value of *E*_t_ with a hybrid shielding gas in 90% Ar + 10% N_2_ is slightly greater than pure argon, but the ip value is almost unchanged. Furthermore, the value of (*E*_t_ − *E*_f_), which shows the passive region, is increased with the addition of nitrogen in the shielding gas. The above results show that, with the addition of nitrogen, the value of ip decreases, the resistance of the passive film to pitting corrosion is improved and the stability increases. Among them, pure nitrogen as a shielding gas has an excellent pitting resistance property. Kyröläinen and Lukkari [33] and the Outokumpu Welding Handbook (Outokumpu, 2010) have noted that a low austenite content can lead to nitride precipitation, which has a negative effect on weld corrosion properties and toughness [34].

**Figure 15:**
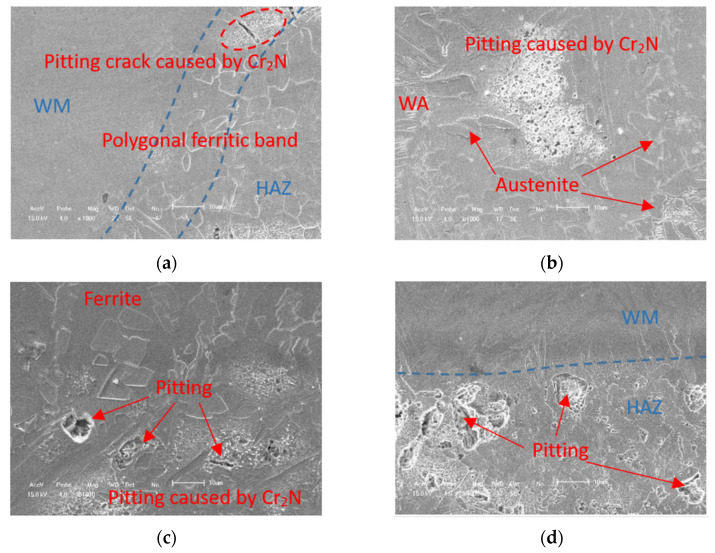
SEM images of selected corroded regions in the DSS joint after potentiostatic test: (**a**,**c**) HAZ near the WM and (**b**,**d**) HAZ near the BM.

Figure 15 demonstrate the SEM images of the pitting morphology formed on the surface of the DSS welds welded in four different shielding gas conditions after anodic polarization tests. It is obvious that the pitting corrosion degree in the HAZ was much more severe than in the WM and BM, so the pitting resistance of the whole welded joint was predominated by the HAZ.

In HAZ, pitting tends to be on the polygonal ferrite band near the fusion line (Figure 15a) and on the boundaries of austenite and ferrite. The reason is that the lean carbon and high a-phase content of the polygonal ferrite zone leads to the precipitation of Cr_2_N. As the nucleation point, Cr_2_N both causes the concentration of corrosion stress and diffuses around it, resulting in corrosion pits (Figure 15c,d).

Therefore, it is concluded that the increase in nitrogen content in the shielding gas can significantly promote the transformation of austenite, increase the solubility of Cr_2_N, reduce the width of the chromium-poor zone and increase the overall corrosion resistance of the weld.

## 4. Conclusions

1. The addition of nitrogen in the shielding gas can increase the austenite content in the weld zone to approximately 51.6% higher than that of pure argon (the nitrogen shielded is 51.6%, the pure argon shielded is 32.2%), but the transformation type of the ferrite–austenite is not affected significantly;
2. The increase in nitrogen content in the shielding gas does not affect the strength of the joint, and the base metal is still the weakest part of the joint;
3. The evaporation loss of nitrogen in the weld pool means that it is easy to form harmful phases, such as an Fe-Cr intermetallic compound in the weld, which is not conducive to the impact toughness of the weld;
4. The addition of nitrogen in the shielding gas is beneficial to austenite regeneration during solidification. The pitting corrosion resistance of the four kinds of welds is not as good as that of base metal; The heat affected zone has poor corrosion resistance. The weld with pure nitrogen protection has the highest Et value and the best corrosion resistance;
5. There is a carbon-poor ferrite band near the fusion line in the HAZ. Due to the fact that the solubility of nitrogen in ferrite decreases rapidly with the decrease in temperature, the supersaturated nitrogen combines with chromium to form chromium nitride precipitation. The precipitation of Cr_2_N results in partial Cr depletion, which is the main reason for the weak pitting resistance of HAZ.

## Figures and Tables

**Table 1:** Specifications of RCL-C6000 fiber laser machine.

Parameter (Unit)	Value
Rated power (W)	6000
Wavelength (nm)	1075–1085
Divergence angle (Rad)	<0.1
Operation mode	Continuous wave
Fiber core diameter (μm)	100

**Table 2:** Chemical composition of base metal and filler metals (wt.%).

Materials	C	Si	Mn	P	S	Cr	Ni	Mo	N	Cu	Co	Nb
Base metal	0.023	0.59	4.9	0.0199	0.001	21.5	1.62	0.26	0.21	0.24	0.025	-
Filler metal	0.012	0.35	1.59	0.015	0.001	22.56	8.62	3.05	0.15	0.06	0.049	0.002

**Table 3:** Mechanical properties of S32101 duplex stainless steel.

Tensile Strength (MPa)		Yield Strength (MPa)		Elongation (%)	Hardness HB	Impact (J)	Ferrite Content (%)
25 °C	130 °C	25 °C	130 °C				
703	602	453	371	49	207	98	56.5

**Table 4:** Welding parameters and composition of shielding gas.

Specimen No.	Layers	Focal Spot Diameter (mm)	Wire Speed (m/min)	Laser Power (kW)	Speed (m/min)	Shielding Gas and Flow Rate (L/min)
A	Root	5	3.2	5000	0.6	Pure Ar, 25
	Filling/finishing	5	2.6	5000	0.6	
B	Root	5	3.2	5000	0.6	90%Ar + 10%N_2_, 25
	Filling/finishing	5	2.6	5000	0.6	
C	Root	5	3.2	5000	0.6	50%N_2_ + 50%Ar, 25
	Filling/finishing	5	2.6	5000	0.6	
D	Root	5	3.2	5000	0.6	Pure N_2_, 25
	Filling/finishing	5	2.6	5000	0.6	

**Table 5:** Results of tensile tests.

No.	Tensile Strength Rm/MPa	Yield Strength Rp0.2/MPa	Elongation/%	Fracture Location
A	749	589	28.5	BM
B	721	581	30.5	BM
C	747	592	26	BM
D	748	586	26.5	BM
BM	766	608	32.5	BM

**Table 6:** EDS results of points shown in Figure 13a (at.%).

Weld	Points	Fe	Cr	Ni	Mn	Mo	Si
A	1	65.494	22.793	7.332	2.448	1.056	0.877
	2	67.624	21.552	4.156	3.889	0.828	1.951
B	1	66.004	22.154	7.296	3.058	0.513	0.975
	2	67.786	21.364	4.514	3.891	1.447	0.998
C	1	64.691	23.269	6.803	3.043	0.293	1.901
	2	66.826	22.416	3.115	4.168	1.096	2.379
D	1	64.574	23.763	6.771	2.448	0.785	1.692
	2	67.872	22.310	3.350	4.196	1.048	1.224

**Table 7:** Electrochemical corrosion parameters after anodic polarization tests.

Shielding Gas	*I*_corr_ μA/cm^2^	*E*_corr_ mV_SCE_	*i*_p_ μA/cm^2^	*E*_t_ mV_SCE_	*E*_t_ − *E*_f_ mV
A	151	3297	52	357	528
B	145	3056	50	348	521
C	74	2756	36	799	1002
D	68	2731	33	847	1010
BM	67	2689	34	824	919
